# Anti-vascular endothelial growth factor dosing frequency and visual outcomes in macular oedema following branch retinal vein occlusion

**DOI:** 10.1038/s41433-023-02527-7

**Published:** 2023-05-08

**Authors:** Yasha S. Modi, Lediana Goduni, Hadi Moini, Andrea Gibson, Nick Boucher, Genevieve Lucas, Dilsher S. Dhoot

**Affiliations:** 1 New York University Langone Health, New York, NY USA; 2 Regeneron Pharmaceuticals, Inc., Tarrytown, NY USA; 3 Vestrum Health, Naperville, IL USA; 4 California Retina Consultants, Santa Barbara, CA USA; 5 Retina Consultants of America, Santa Barbara, CA USA; 6 Present Address: Ocular Therapeutics, Bedford, MA USA

**Keywords:** Retinal diseases, Drug therapy

## Abstract

**Objectives:**

To determine the relationship between treatment frequency with intravitreal anti-vascular endothelial growth factor (anti-VEGF) agents and visual acuity (VA) outcomes in eyes with macular oedema (MO) secondary to branch retinal vein occlusion (BRVO) in US clinical practice.

**Methods:**

Study eyes that initiated anti-VEGF injections between January 2012 and May 2016 were followed for ≥1 year in a retrospective analysis of medical records (Vestrum Health database). Eyes were analysed in 2 cohorts by treatment duration (years 1 and 2) and then in 2 subcohorts by injection frequency (≤6 or ≥7 injections/year).

**Results:**

Among 3099 eyes with MO secondary to BRVO, 1197 (38.6%) received ≤6 injections (mean injections, 4.6; baseline mean VA, 53 letters) and 1902 (61.4%) received ≥7 injections through 1 year (mean injections, 8.8; baseline mean VA, 52 letters). At year 1, mean VA gain from baseline was 10.4 versus 13.9 letters in eyes receiving ≤6 versus ≥7 injections (*p* < 0.001). At year 2, mean VA in eyes receiving ≤6 (*n* = 42) versus ≥7 injections (*n* = 227) was 64 versus 68 letters, respectively (*p* = 0.19). Mean VA change between the start and end of year 2 in eyes receiving ≥7 injections in year 1 and ≤6 in year 2 differed significantly from that of eyes receiving ≥7 injections in both years (–3.0 vs 0.7 letters, respectively; *p* < 0.001).

**Conclusions:**

In routine clinical practice, more frequent dosing with anti-VEGF agents was associated with greater visual benefits in eyes with MO secondary to BRVO.

## Introduction

Retinal vein occlusion is the second most common retinal vascular disease and can result in profound vision loss in affected patients [1, 2]. Despite many proposed interventions, there are no definitive treatments for this condition [2]. Management is mainly directed at secondary complications of retinal vein occlusion that affect vision, such as macular oedema (MO) and retinal neovascularisation [3, 4].

Anti-vascular endothelial growth factor (anti-VEGF) agents are the mainstay treatment of MO secondary to branch retinal vein occlusion (BRVO) [1, 3]. Multicentre, randomised, pivotal trials demonstrated the efficacy and safety of intravitreal ranibizumab 0.5 mg (BRAVO) and aflibercept 2.0 mg (VIBRANT) in patients with MO secondary to BRVO [5, 6]. In these trials, patients were initially treated with monthly anti-VEGF injections for 6 months followed by monthly monitoring and pro re nata (BRAVO) or every-8-week (VIBRANT) dosing for an additional 6 months. These landmark studies demonstrated significant visual gains at 6 months (18.3 and 17.0 letters following mean of 5.7 and 5.7 injections, respectively) and 12 months (18.3 and 17.1 letters following mean of 8.4 and 9.0 injections, respectively) in BRAVO and VIBRANT, respectively [7, 8]. Off-label bevacizumab was also evaluated for the treatment of MO secondary to BRVO and demonstrated efficacy in improving vision over 6 months (14.2 letters following approximately 6 injections) [9].

Despite robust evidence supporting the outcomes of anti-VEGF therapy in clinical trials, information about the outcomes of patients with BRVO in real-world clinical settings is currently limited. The heterogeneity of a real-world patient population [10] and variability of actual clinical practice based on local regulations and treatment access are not fully represented by trial data. Currently, the outcomes of MO secondary to BRVO following anti-VEGF therapy in real-world settings have been evaluated only by a few small and mostly non-US studies [11–14].

In the current study, we used a large real-world database of patients with MO secondary to BRVO in the USA to assess the relationships between visual acuity (VA), anatomic outcomes, and treatment frequency.

## Materials and methods

### Data source

Data were taken from de-identified electronic medical records of patients with MO secondary to BRVO from 251 retina specialists at 54 private clinics in the USA, in the Vestrum Health Treatment and Outcomes database (Vestrum Health, Naperville, Illinois, USA). Demographics, procedures, diagnosis, medications, and treatment outcomes data were reported in these records. Structured language queries were used to extract data from the database. Institutional Review Board approval was not required as data collection included only de-identified electronic health records, and did not affect or influence patient treatment.

### Study population

The study population comprised eyes that were diagnosed with MO secondary to BRVO and administered their first (index) anti-VEGF injection between 1 January 2012 and 31 May 2016. Eyes were included in the study if they had a VA reading on the index date, at month 12, and at least once during each quarter of the study period. Eyes were excluded if there was a break from treatment for longer than 11 months at any point in the 24 months following the index date or if sex identification was not recorded. For consistency and to ensure comparable results, all Snellen VA measurements for an individual patient were required to use the same methodology; these were calculated using the formula for Early Treatment Diabetic Retinopathy Study (ETDRS) letters = 85 + 50 × log(Snellen fraction) [15].

### Observation period

All eyes were observed for 12–24 months from baseline. The observation period began on 1 January 2012 and ended on 31 May 2018, inclusive of all eyes.

### Cohorts

Eyes were analysed in 2 cohorts: year 1 cohort (eyes that were treated for 1 year) and year 2 cohort (eyes that were treated for 2 years). Each of these cohorts was further divided into 2 subcohorts based on whether ≤6 injections or ≥7 injections were administered per year, hereafter referred to as the ≤6-injections and ≥7-injections subcohorts.

### Statistical methods

Descriptive statistics were calculated for the year 1 and year 2 cohorts to identify changes in injection frequency and ETDRS letters over time. Paired *t* tests were used to determine whether the changes in injection frequency and ETDRS letters over time were significant. Independent *t* tests assuming unequal variance were used to determine whether the differences in injection frequency and change in ETDRS letters between cohorts were significant. Calculations were performed using Microsoft Excel. *P* < 0.05 was considered statistically significant.

## Results

### Disposition and patient characteristics

**Fig. 1:**
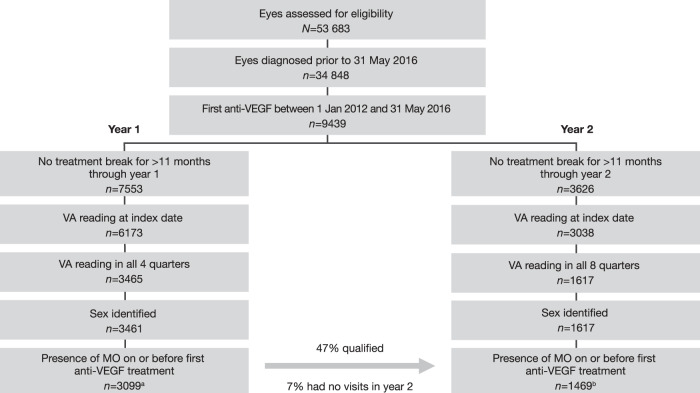
Disposition of eyes with MO secondary to BRVO. ^a^Eyes from 3070 patients. ^b^Eyes from 1457 patients. BRVO branch retinal vein occlusion, MO macular oedema, VA visual acuity, VEGF vascular endothelial growth factor.

Overall, 53,683 eyes with MO secondary to BRVO were assessed for eligibility (Fig. 1). Of these, 9439 received their initial anti-VEGF injection between 1 January 2012 and 31 May 2016 and had a VA reading on the same day as the index injection. After excluding eyes that did not have all required quarterly VA readings, those without sex identification, and those who had treatment breaks longer than 11 months during follow-up, 3099 and 1469 eyes were included in the year 1 and year 2 cohorts, respectively. In total, 47% (*n* = 1469) of eyes in the year 1 cohort qualified for inclusion in the year 2 cohort. Seven percent (*n* = 222) of eyes in the year 1 cohort did not have any visits in year 2, and the remainder were excluded due to incomplete VA readings at baseline or every quarter, or treatment breaks longer than 11 months over the 2 years of follow-up.

In the year 1 cohort, 12% (*n* = 387) of eyes received aflibercept, 30% (*n* = 937) received bevacizumab, 31% (*n* = 972) received ranibizumab, and 26% (*n* = 803) received more than one anti-VEGF therapy. In the year 2 cohort, 15% (*n* = 217) of eyes received aflibercept, 30% (*n* = 436) received bevacizumab, 28% (*n* = 413) received ranibizumab, and 27% (*n* = 403) received more than one anti-VEGF therapy. Injection frequencies were similar between anti-VEGF treatment types used for both year 1 and year 2 (Supplementary Table 1).

Regardless of anti-VEGF treatment type, eyes in the year 1 cohort (*n* = 3099) received a mean of 7.2 (median, 7.0) injections during year 1, and were divided into 2 subcohorts according to receipt of ≤6 injections (1197/3099; 38.6%) or ≥7 injections (1902/3099; 61.4%) through year 1. Baseline characteristics, including baseline vision, were similar between the ≤6-injections and ≥7-injections subcohorts (Table 1). In year 1, 24.6% (295/1197) of eyes in the ≤6-injections subcohort and 21.1% (402/1902) of eyes in the ≥7-injections subcohort had received concomitant steroid and/or laser therapy, with a mean (range) of 4.3 (2–6) treatments and 9.5 (7–13) treatments, respectively (Table 1).

**Table 1:** Demographic and baseline characteristics of patients with MO secondary to BRVO in the year 1 cohort by injection frequency during year 1.

Characteristic	Total (*N* = 3099)	Injection subcohort	
		≤6 injections (*n* = 1197)	≥7 injections (*n* = 1902)
Age, mean, years	72	73	71
Female, *n* (%)	1678 (54)	653 (55)	1025 (54)
VA, letters			
Mean	52	53	52
Median	60	60	59
VA subgroup, *n* (%)			
≥20/40	718 (23)	302 (25)	416 (22)
<20/40–20/100	1487 (48)	557 (47)	930 (49)
<20/100–20/200	467 (15)	175 (15)	292 (15)
<20/200	427 (14)	163 (14)	264 (14)
Eyes receiving steroids and/or laser in year 1, *n* (%)	697 (22)	295 (25)	402 (21)
Number of such treatments, mean (range)	7.0 (2–13)	4.3 (2–6)	9.5 (7–13)

VA is reported in approximate Snellen equivalent converted from ETDRS letters.

*BRVO* branch retinal vein occlusion, *ETDRS* Early Treatment Diabetic Retinopathy Study, *MO* macular oedema, *VA* visual acuity.

### Year 1 outcomes

**Fig. 2:**
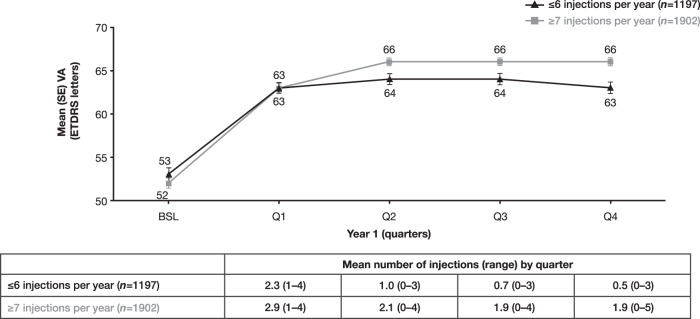
Mean VA through year 1 in eyes with MO secondary to BRVO in the ≤6-injections and ≥7-injections subcohorts. BRVO branch retinal vein occlusion, BSL baseline, ETDRS Early Treatment Diabetic Retinopathy Study, MO macular oedema, Q quarter, SE standard error, VA visual acuity.

For eyes that were treated for ≥1 year, those in the ≤6-injections subcohort received a mean of 4.6 (range, 2–6) injections and eyes in the ≥7-injections subcohort received a mean of 8.8 (range, 7–14) injections in year 1. In the ≤6-injections subcohort, mean VA increased from 53 letters at baseline to 63 letters after 1 year of treatment (Fig. 2), and in the ≥7-injections subcohort, from 52 letters at baseline to 66 letters at year 1. Mean VA gain from baseline was significantly lower in the ≤6-injections subcohort than the ≥7-injections subcohort at year 1 (10.4 vs 13.9; *p* < 0.001). Consistent with this finding, a sensitivity analysis indicated similarly lower mean VA gains in eyes that received ≤6-injections compared with those that received ≥7-injections among a subgroup of eyes that did not receive steroids or laser (*n* = 2402; 11.1 vs 14.3, *p* < 0.001), as well as those that received steroids and/or laser (*n* = 697; 8.5 vs 12.3, *p* = 0.041) (Supplementary Fig. 1). Eyes that received laser treatment received a similar mean number of anti-VEGF injections during year 1 as eyes that did not receive laser (7.0 vs 7.3 injections).

**Fig. 3:**
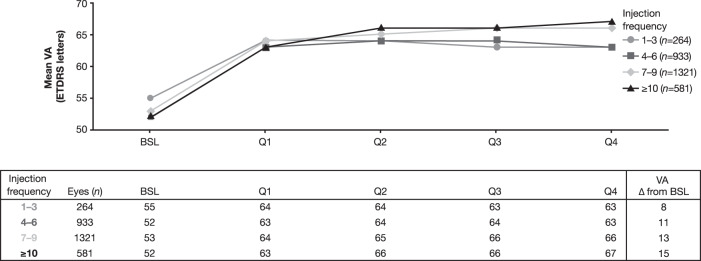
Mean VA change through year 1 by injection frequency during year 1 in eyes with MO secondary to BRVO. Analysis included eyes with available VA measurements through Year 1. BRVO branch retinal vein occlusion, BSL baseline, ETDRS Early Treatment Diabetic Retinopathy Study, MO macular oedema, Q quarter, VA visual acuity.

To assess the relationship between the frequency of treatment and visual and anatomic outcomes, eyes were further stratified into 4 subcohorts based on injection frequency: 1–3, 4–6, 7–9, and ≥10 injections over year 1. Across the 4 subcohorts of eyes for which VA was available, mean VA ranged from 52–55 letters at baseline and improved to 63–67 letters at year 1 (Fig. 3). Overall and on average, VA gain increased with increasing injection frequency. In a subset of these eyes with both VA and foveal thickness measurements available, a trend showing improved (thinner) foveal thickness with increasing injection frequency was also observed, with eyes in the 1–3-injections subcohort being outliers in this trend as their visual and anatomic outcomes were relatively better than those in the 4–6 injection subcohort (Supplementary Fig. 2). In Year 1, 3.9% (121/3099) of eyes received a mean (range) of 2.7 (2–4) injections in Q1 with no additional injections in Q2, Q3, or Q4; these eyes were all in the ≤6-injections subcohort.

### Year 2 outcomes

**Fig. 4:**
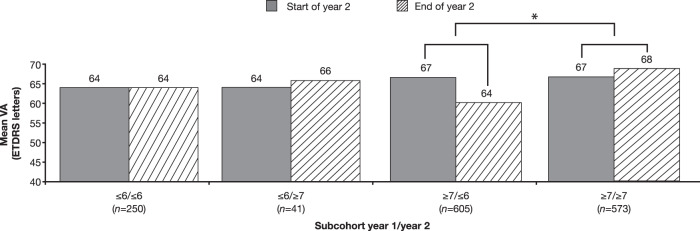
Mean VA at the start and end of year 2 by injection frequency in years 1 and 2 in eyes with MO secondary to BRVO. *P < 0.001 compared with the change in the ≥7/≥7-injections subcohort. BRVO branch retinal vein occlusion, ETDRS Early Treatment Diabetic Retinopathy Study, MO macular oedema, VA visual acuity.

Eyes that received ≤6 injections in year 1 and also subsequently received ≤6 injections in year 2 (*n* = 250) received a mean of 5.2 and 4.2 injections in years 1 and 2, respectively. At the start of year 2, mean VA of these eyes was 64 letters, and this score was maintained through the end of year 2 (Fig. 4). Eyes that received ≤6 injections in year 1 and ≥7 injections in year 2 (*n* = 41) were administered a mean of 5.2 and 7.9 injections in years 1 and 2, respectively. These eyes started year 2 with a mean VA of 64 letters and ended year 2 with a mean VA of 66 letters (*p* = 0.69).

Eyes that received ≥7 injections in year 1 and subsequently ≤6 injections in year 2 (*n* = 605) received a mean of 7.5 and 4.6 injections in years 1 and 2, respectively. These eyes started year 2 with a mean VA of 67 letters and ended year 2 with a mean of 64 letters, showing an average loss of 3 letters (Fig. 4). Eyes that received ≥7 injections in year 1 and continued to receive ≥7 injections in year 2 (*n* = 573) received a mean of 9.7 and 8.5 injections in years 1 and 2, respectively. These eyes started year 2 with a mean of 67 letters and ended year 2 with a mean of 68 letters, gaining an average of 0.7 letters. The change in mean VA between the start and end of year 2 in the subcohort of eyes that received ≤6 injections in year 2 and ≥7 injections in year 1 differed significantly from the subcohort of eyes that received ≥7 injections in both years 1 and 2 (–3 vs +1 letter; *p* < 0.001). It is important to note that patients who received ≥7 injections in years 1 and 2 avoided loss of approximately one line of VA compared with those that received ≥7 injections in year 1 and ≤6 injections in year 2 with an additional three anti-VEGF injections per year.

In a subset of eyes in the ≤6-injections or ≥7-injections subcohorts for both years 1 and 2, and for which measurements for both VA and foveal thickness at baseline and all 8 quarters through year 2 were available, there was a trend toward increased VA and decreased foveal thickness with increasing injection frequency (Supplementary Fig. 3).

Eyes that received laser treatment received a similar mean number of anti-VEGF injections during year 2 as eyes that that did not receive laser (14.3 vs 14.4 injections).

### Annual trend in treatment frequency during year 1

The mean number of anti-VEGF injections given during year 1 was similar between 2012 and 2016 for both the ≤6-injections subcohort (4.4–4.7 injections) and the ≥7-injections subcohort (8.6–8.9 injections) (Supplementary Fig. 4). However, numerically greater proportions of eyes received ≥7 injections during year 1 between 2014 and 2016 (63–65%) than in either 2012 (41%) or 2013 (51%) (Supplementary Fig. 4).

## Discussion

This real-world analysis of eyes with MO secondary to BRVO demonstrated an association between anti-VEGF injection frequency and visual gains over 2 years in routine clinical practice in the USA. Eyes receiving ≥7 injections per year demonstrated greater VA gains than those receiving ≤6 injections per year, and both groups had similar baseline VA. The corresponding changes in foveal thickness were consistent with the changes seen in VA over 2 years. When further stratified by injection frequency over year 1, visual gains appeared to increase with increasing frequency of injections, with a ceiling effect at 7–9 injections, after which more frequent injections (≥10 injections) did not demonstrate an incremental VA benefit. Collectively, these findings suggest eyes that received at least 7 injections per year over the first and second year achieved greater visual gains than those receiving less frequent injections.

Our findings in eyes receiving ≤6 injections in year 1 (10.4-letter gain with a mean of 4.6 injections) are in agreement with other real-world analyses of BRVO populations including the LUMINOUS study (*n* = 189) conducted across 42 countries (excluding the USA) (11.9-letter gain with a mean of 5 injections) and the OCEAN study (*n* = 204) conducted in Germany (13.1-letter gain with a mean of 4.9 injections) [11, 12]. A UK-based study (*n* = 100) reported no statistically significant visual gains with fewer injections (approximately 4-letter gain with a mean of 3.3 injections) [13]. In the present study, VA gains in eyes receiving ≥7 injections in year 1 (13.9-letter gain with a mean of 8.8 injections) were higher than those reported by the ECHO study (*n* = 95) conducted in the USA (approximately 10-letter gain with a mean of 7.1 injections) [14] but similar to those observed in a subset of LUMINOUS patients who received more frequent injections (13.6-letter gain with 6–9 injections vs 11.7-letter gain with 2–5 injections and 3.6-letter gain with 1 injection) [11]. Collectively, these studies lend support to the observation that more frequent injections provide greater visual benefits. Although visual gains in real-world studies, including our current analysis, were significant, they appeared relatively smaller than those reported in the BRAVO and VIBRANT clinical trials (18.3- and 17.1-letter gains with a mean of 8.4 and 9.0 injections, respectively) [7, 8]. Taken altogether, these findings suggest that while injection frequency is a key modifiable factor to attain optimal visual outcomes, other variables such as patient population characteristics in real-world clinical practice compared with those in the clinical trials may account for some of the observed differences.

The results of our study must be interpreted with caution. The treatment paradigms used by treating clinicians were not taken into consideration, as the Vestrum Health database did not capture this information. In routine clinical practice, patients may start out on one therapy and switch to another based on initial response and individualised treatment. This analysis was designed to assess only injection frequency, not visit frequency. As a result, it is difficult to ascertain whether it was the increased injection frequency alone that contributed to improved outcomes, or also the frequent evaluations and follow-ups that accompanied those injections. Of note, eyes were excluded from the year 2 analysis if they did not meet the inclusion criteria (VA reading on prespecified dates and at least once during each quarter, as well as treatment breaks ≥11 months), which favoured retention of eyes in the study that received more frequent injections. This study was designed agnostic to anti-VEGF treatment type and did not intend to assess the superiority or inferiority of one anti-VEGF treatment agent relative to another, and hence patient data by anti-VEGF treatment type were not collected. Additional limitations include the retrospective nature of the study design, the conversion of Snellen acuity to ETDRS letters [15], the potential confounding impact of cataract formation due to concomitant use of steroids, and the potential effect of macular ischaemia on visual outcomes. Approximately 22% of eyes received steroids and/or laser; excluding such eyes would have reduced the size and scope of our real-world study. A strength of our analysis is the large sample size, which exceeds previously published studies.

In this analysis of real-world data, more frequent dosing with anti-VEGF therapy over 2 years was associated with greater VA gains. This information may provide important insight to the treating clinician for optimising treatment frequency to maximise visual outcomes in eyes with MO secondary to BRVO.

## Summary

### What was known before

- Evidence from clinical trials established the efficacy and safety of anti-vascular endothelial growth factor (anti-VEGF) therapy for patients with macular oedema (MO) secondary to branch retinal vein occlusion (BRVO). However, there is limited information regarding the outcomes of these patients in real-world clinical settings.
- In addition, the relationship between intravitreal anti-VEGF treatment frequency and visual acuity (VA) outcomes in eyes with MO secondary to BRVO in US clinical practice is not fully understood.

### What this study adds

- This real-world analysis showed that eyes with MO secondary to BRVO receiving ≥7 injections per year demonstrated greater VA gains than those receiving ≤6 injections per year over a period of 2 years.
- This information may provide important insight for physicians on how treatment frequency can be used to maximise visual outcomes in eyes with MO secondary to BRVO.

### Supplementary information

Supplementary information

## Data Availability

All relevant data are included in the manuscript and supplement.
