# Supplementary material for: Anti-vascular endothelial growth factor dosing frequency and visual outcomes in macular oedema following branch retinal vein occlusion
Source: Eye (Lond). 2023 May 8;37(16):3423–8. doi: 10.1038/s41433-023-02527-7 (PMC10630437; doi:10.1038/s41433-023-02527-7)
Supplement: Supplementary file 1 — Supplementary information [file 41433_2023_2527_MOESM1_ESM.docx]

# Supplementary information

## Supplementary Fig 1. Mean VA through year 1 for (A) eyes receiving no steroids or laser and (B) eyes receiving steroids and/or laser with MO secondary to BRVO


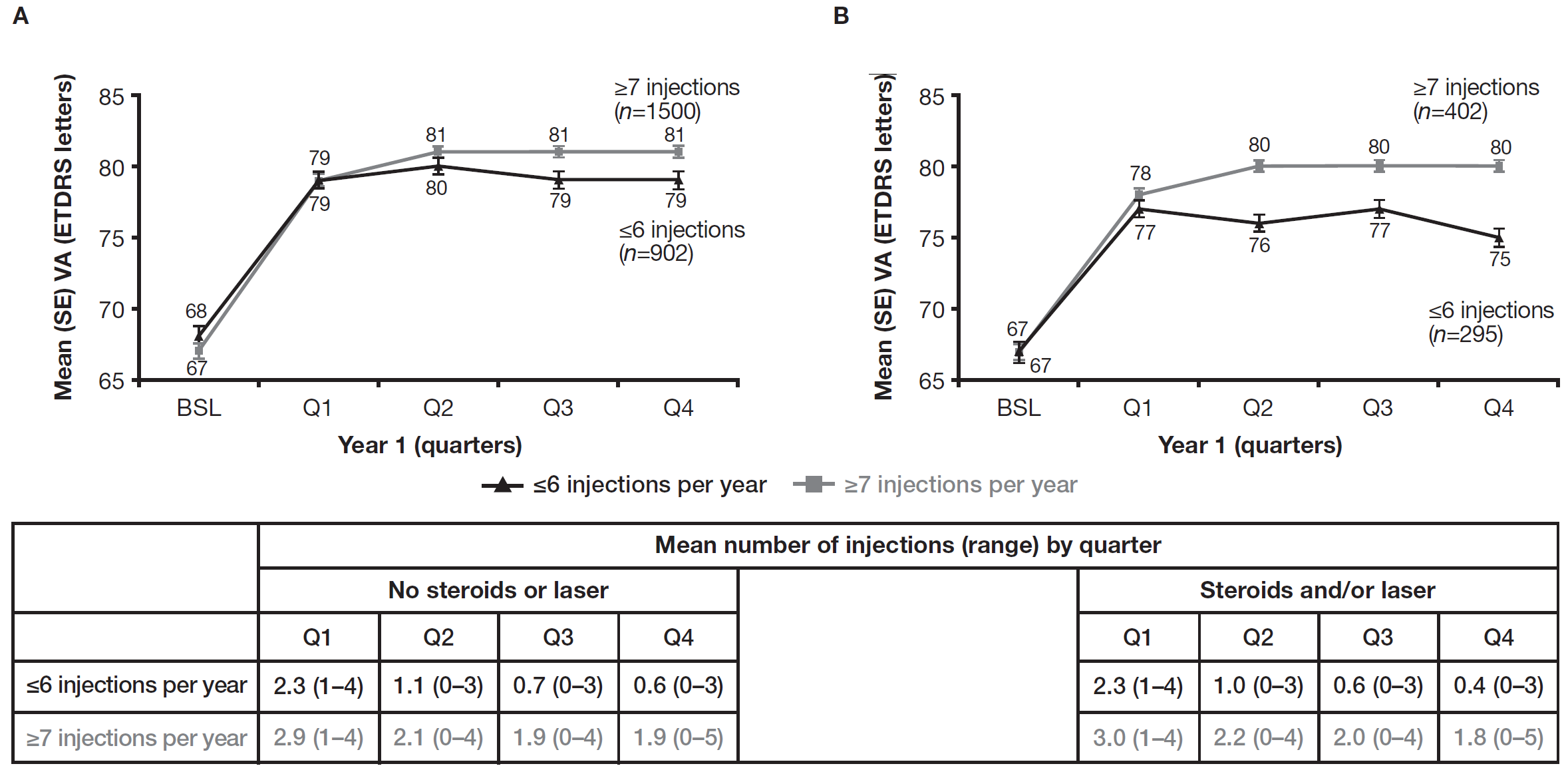


BRVO, branch retinal vein occlusion; BSL, baseline; ETDRS, Early Treatment Diabetic Retinopathy Study; MO, macular oedema; Q, quarter; SE, standard error; VA, visual acuity.

## Supplementary Fig. 2. Mean VA and mean foveal thickness through year 1 by injection frequency in year 1 in eyes with MO secondary to BRVO


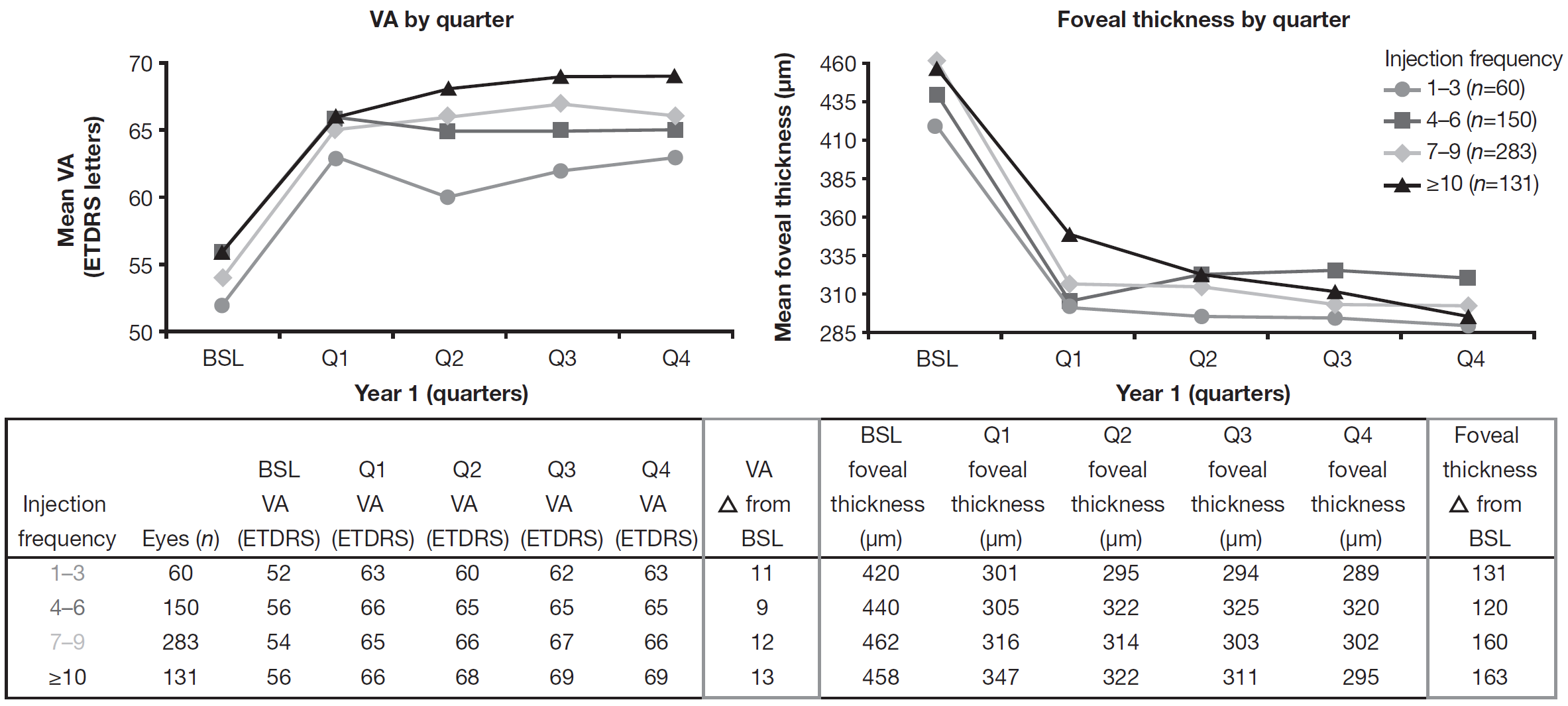


Analysis included eyes with available VA and foveal thickness measurements through Year 1.

BRVO, branch retinal vein occlusion; BSL, baseline; ETDRS, Early Treatment Diabetic Retinopathy Study; MO, macular oedema; Q, quarter; VA, visual acuity.

## Supplementary Fig. 3. Mean VA and mean foveal thickness through year 2 by injection frequency over years 1 and 2 in eyes with MO secondary to BRVO in the ≤6-injections and ≥7-injections subcohorts


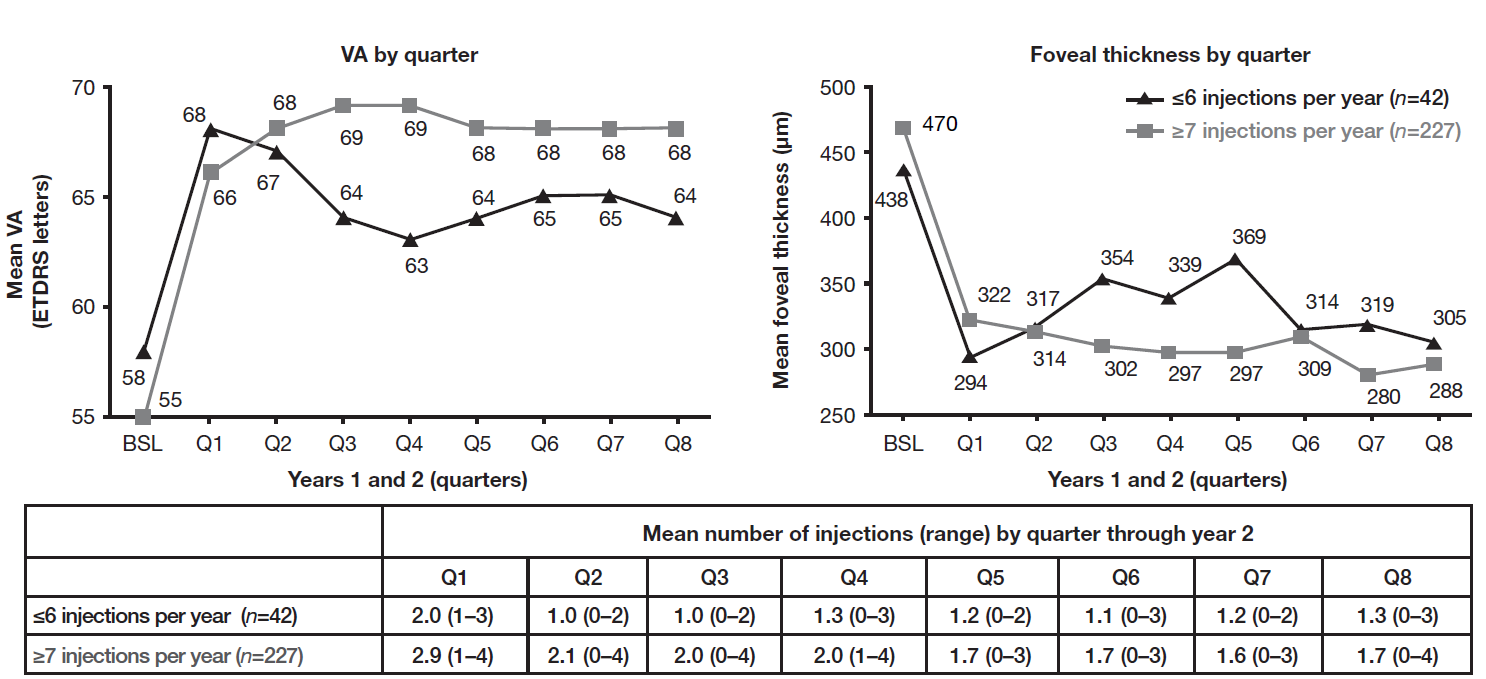


Analysis included eyes with VA and foveal thickness measurements at BSL and all 8 quarters through Year 2.
BRVO, branch retinal vein occlusion; BSL, baseline; ETDRS, Early Treatment Diabetic Retinopathy Study; MO, macular oedema; Q, quarter; VA, visual acuity.

## Supplementary Fig. 4. (A) Mean number of injections per year by index treatment year and (B) distribution of eyes that received ≤6 injections and ≥7 injections during year 1 by index treatment year


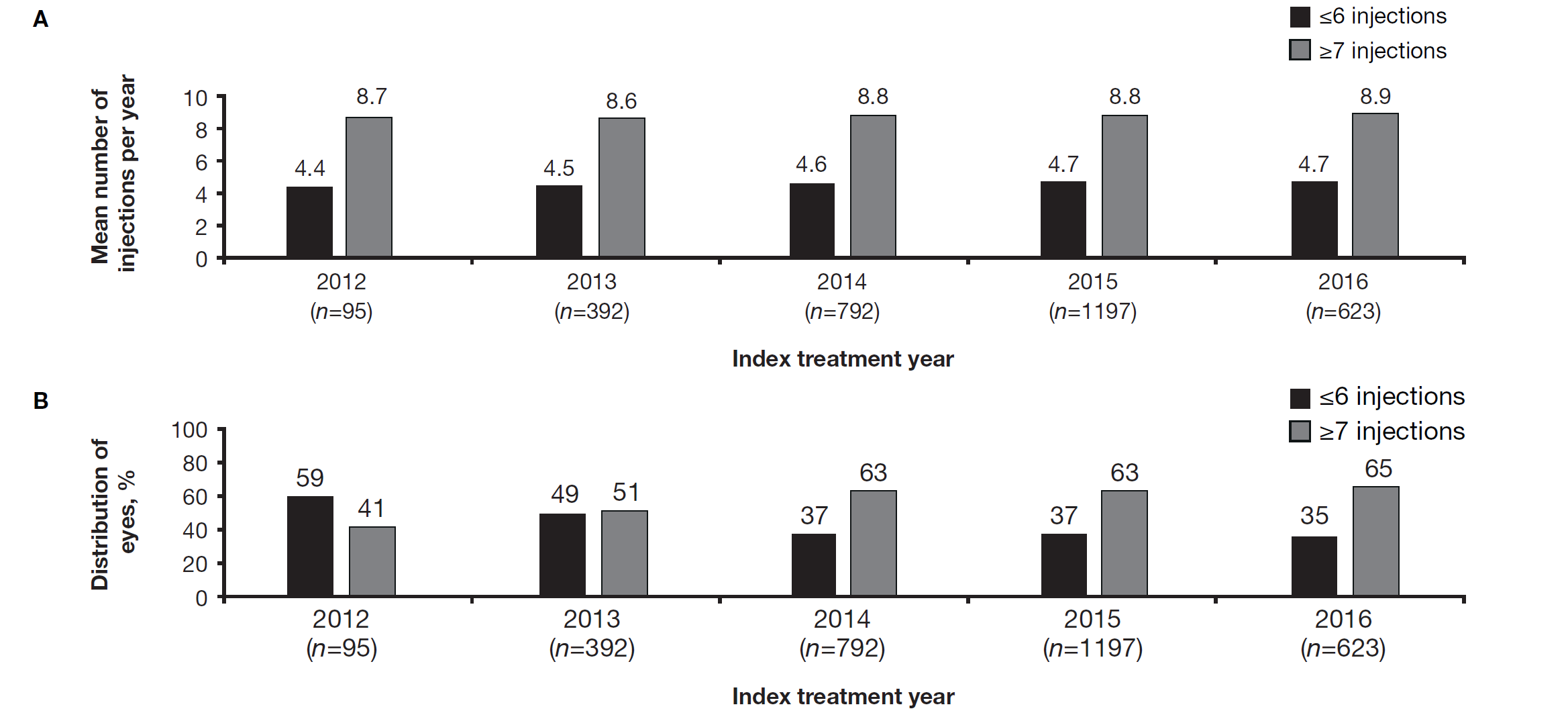


Mean number of injections were determined as the average number of injections administered in the 12 months after index treatment date. BRVO, branch retinal vein occlusion; MO, macular oedema.

## Supplementary Table 1. Injection frequency of eyes in year 1 and year 2 by anti-VEGF type

|  | **Anti-VEGF treatment type** | | | | |  |
| --- | --- | --- | --- | --- | --- | --- |
| **Year 1** | **Bevacizumab** | **Aflibercept** | **Ranibizumab** | **Mixed treatment** | **Total** | |
| Eyes, *n* (%) | 937 (30) | 387 (12) | 972 (31) | 803 (26) | 3099 (100) | |
| Injections, mean | 6.62 | 6.51 | 7.04 | 8.36 | 7.19 | |
| **Year 2** |  |  |  |  |  | |
| Eyes, *n* (%) | 436 (30) | 217 (15) | 413 (28) | 403 (27) | 1469 (100) | |
| Injections year 1, mean | 8.25 | 8.26 | 8.19 | 8.38 | 8.27 | |
| Injections year 2, mean | 6.16 | 6.34 | 6.02 | 6.16 | 6.15 | |

Mean number of injections were determined as the average number of injections administered in the 12 months after index treatment date.
